# The influence of origin and race location on performance in IRONMAN^®^ age group triathletes

**DOI:** 10.1371/journal.pone.0315064

**Published:** 2024-12-09

**Authors:** Beat Knechtle, David Valero, Elias Villiger, Mabliny Thuany, Pantelis T. Nikolaidis, Ivan Cuk, Marilia Santos Andrade, Pedro Forte, Lorin Braschler, Thomas Rosemann, Katja Weiss

**Affiliations:** 1 Medbase St. Gallen Am Vadianplatz, St. Gallen, Switzerland; 2 Institute of Primary Care, University of Zurich, Zurich, Switzerland; 3 Ultra Sports Science Foundation, Pierre-Benite, France; 4 Department of Physical Education, State University of Para, Pará, Brazil; 5 School of Health and Caring Sciences, University of West Attica, Athens, Greece; 6 Faculty of Sport and Physical Education, University of Belgrade, Belgrade, Serbia; 7 Department of Physiology, Federal University of Sao Paulo, Sao Paulo, Brazil; 8 CI-ISCE, Higher Institute of Educational Sciences of the Douro, Penafiel, Portugal; 9 Department of Sports Sciences, Insituto Politécnico de Bragança, Bragança, Portugal; 10 Research Center in Sports Health and Human Development, Covilhã, Portugal; 11 Faculty of Medicine, University of Bern, Bern, Switzerland; Geisinger Health System, UNITED STATES OF AMERICA

## Abstract

**Background:**

The IRONMAN^®^ (IM) triathlon is a popular multi-sport, where age group athletes often strive to qualify for the IM World Championship in Hawaii. The aim of the present study was to investigate the location of the fastest IM racecourses for age group IM triathletes. This knowledge will help IM age group triathletes find the best racecourse, considering their strengths and weaknesses, to qualify.

**Objective:**

To determine the fastest IM racecourse for age group IM triathletes using descriptive and predictive statistical methods.

**Methods:**

We collected and analyzed 677,702 age group IM finishers’ records from 228 countries participating in 444 IM competitions held between 2002 and 2022 across 66 event locations. Locations were ranked by average race speed (performance), and countries were sorted by number of records in the sample (participation). A predictive model was built with race finish time as the predicted variable and the triathlete’s gender, age group, country of origin, event location, average air, and water temperatures in each location as predictors. The model was trained with 75% of the available data and was validated against the remaining 25%. Several model interpretability tools were used to explore how each predictor contributed to the model’s predictive power, from which we intended to infer whether one or more predictors were more important than the others.

**Results:**

The average race speed ranking showed IM Vitoria-Gasteiz (1 race only), IM Copenhagen (8 races), IM Hawaii (18 races), IM Tallinn (4 races) and IM Regensburg (2 races) in the first five positions. The XG Boost Regressor model analysis indicated that the IM Hawaii course was the fastest race course and that male athletes aged 35 years and younger were the fastest. Most of the finishers were competing in IM triathlons held in the US, such as IM Wisconsin, IM Florida, IM Lake Placid, IM Arizona, and IM Hawaii, where the IM World Championship took place. However, the fastest average times were achieved in IM Vitoria-Gasteiz, IM Copenhagen, IM Hawaii, IM Tallin, IM Regensburg, IM Brazil Florianopolis, IM Barcelona, or IM Austria with the absolutely fastest race time in IM Hawaii. Most of the successful IM finishers originated from the US, followed by athletes from the UK, Canada, Australia, Germany, and France. The best mean IM race times were achieved by athletes from Austria, Germany, Belgium, Switzerland, Finland, and Denmark. Regarding environmental conditions, the best IM race times were achieved at an air temperature of ∼27°C and a water temperature of ∼24°C.

**Conclusions:**

IM age group athletes who intend to qualify for IM World Championship in IM Hawaii are encouraged to participate in IM Austria, IM Copenhagen, IM Brazil Florianopolis, and/or IM Barcelona in order to achieve a fast race time to qualify for the IM World Championship in IM Hawaii where the top race times were achieved. Most likely these races offer the best ambient temperatures for a fast race time.

## Introduction

The IRONMAN^®^ (IM) triathlon covering 3.8 km swimming, 180 km cycling and 42.195 km running, is one of the most popular triathlon formats [1]. Since the first edition of IM Hawaii, different topics such as training [2], nutrition [3], pacing [4], predictive variables [5], age of peak performance [6], and the determination of the most predictive split discipline [7] led to scientific insights for athletes and coaches in regards to IM race preparation. Most professional and recreational age group IM triathletes want to compete in IM Hawaii as the IM World Championship [8]. Therefore, it would be of great interest for IM age group triathletes to know where the fastest IM racecourse is.

IM age group triathletes need to qualify in an IM race by ranking within their age group (in 5-year intervals). Each IM race offers several slots for each age group category. The goal of many IM age group triathletes is to achieve a new personal best time. For these athletes, the knowledge of the fastest IM racecourse is highly valuable. There are many possibilities to get a slot for IM Hawaii for age group triathletes. Of all the 19 potential possibilities, the first is to win the age group category in a full-distance IM triathlon race [https://blog.irace.ai/how-to-qualify-for-kona-ironman-world-championships]. Considering this information and the knowledge of each athlete’s strengths and weaknesses, such as technical skill in cycling, ability to adapt to different weather conditions, or the number of slots available to compete in IM Hawaii, they could decide in which race to participate to achieve their goal.

To date, there is no knowledge about the predictive factors of racecourses in the IM triathlon races. However, originating from the United States of America (USA) [9, 10] seemed to be a predictive factor for a fast IM race time. An analysis of the IM World Championship IM Hawaii and its qualifying races showed that in 2010, US-American triathletes dominated participation and performance in both IM Hawaii and its qualifiers [8]. A study analyzing 39,706 finishers from 124 countries and competing between 1985 and 2012 in IM Hawaii showed that most finishers originated from the USA [9]. However, another study investigating the pacing of 302,535 IM triathletes competing between 2002 and 2015 in 253 different IM races showed that Germans were the fastest, followed by Australians, Austrians, and Brazilians, where IM triathletes from the USA were not among the fastest [10]. The mentioned studies showed where the fastest IM triathletes originated from, but IM age group triathletes cannot extract from these studies where the fastest IM racecourses are located for their intention. Furthermore, the disparate findings regarding the dominance of US-American or German triathletes need more investigation.

Overall, age group triathletes form the largest group of athletes competing in an IM triathlon [11]. Over the years, IM age group triathletes have improved their performance in IM qualifiers [12] and in IM Hawaii itself [13]. Since IM age group triathletes form the largest group of athletes in any IM triathlon race and these athletes need to qualify for IM Hawaii, it might be of high interest for them to know where the fastest IM racecourses are, in order to choose, considering their strengths and weakness, the best IM race to participate.

Environmental conditions such as water [14] and air temperature [15] have a considerable influence on endurance performance. When different weather parameters (*i*.*e*. air temperature, humidity, wind speed, and solar load) were evaluated in running events of different distances, air temperature showed the highest influence where lower air temperatures of 10°C-17.5°C led to better performances [16]. In marathon running, performance progressively slowed down with increasing air temperature, especially for slower [17] and older [18] runners. Regarding triathlon, an actual study investigating the Olympic distance triathlon showed differences between the split disciplines and the sexes regarding the ambient temperatures. While cycling showed the highest influence on overall race time at low temperatures, running had the highest influence at high temperatures. Regarding the sex, female sex showed a higher influence on overall race time in cycling but less in running when swimming was performed in a river [19]. A study regarding the influence of temperatures on race performance is, however, missing for IM triathletes.

Therefore, the present study aimed to find the fastest racecourses for IM age group triathletes from all IM races held between 2002 and 2022 worldwide. With this knowledge, IM age group triathletes might be able to select a specific IM racecourse to qualify for IM Hawaii. Nonetheless, the optimal selection of a racecourse should be carried out carefully, considering the increased effort and associated financial cost of participation. Since originating from the USA seemed to be a predictive factor for a fast IM race times [9, 10], we hypothesized that most IM age group triathletes would (i) originate from the USA and (ii) would compete in IM races held in the USA. A second aim was to investigate a potential influence of water and air temperatures as well as course profiles on overall race time. We hoped to find (i) the ideal ambient water and air temperatures and (ii) the ideal course profiles in cycling and running to achieve a fast overall race time.

## Methods

### Declarations ethics approval

This study was approved by the Institutional Review Board of Kanton St. Gallen, Switzerland, with a waiver of the requirement for informed consent of the participants as the study involved the analysis of publicly available data (EKSG 01/06/2010). The study was conducted in accordance with recognized ethical standards according to the Declaration of Helsinki adopted in 1964 and revised in 2013.

### Data set and data preparation

The race data was downloaded from the official IRONMAN^®^ website (www.ironman.com) using a Python script (www.python.org). The athletes’ sex, age and country of origin, the event location and year, the partial times (i.e., swimming, running, cycling, and transition times) and the overall race times were thus obtained. We also included the average air and water temperature during these races as provided by the race websites. After extensive clean-up and data pre-processing, a total of 677,702 IM finishers’ records were available for analysis from IM triathletes originating from 228 different countries and competing at 444 races held between 2002 and 2022 in 66 different event IM race locations worldwide. The raw dataset included records of athletes that did not complete the race (DNS–did not start, DNF–did not finish, DQ–disqualified), which were discarded. Further processing of the dataset included harmonization of age group and gender information, checking for data coherence (meaning that the sum of the split times would match the overall race time, no negative or impossible times, etc.), the harmonization of countries of origin of the athletes (discarding records without such information, ensuring all country acronyms corresponded to actual countries, etc.), the harmonization of event locations (we found that some generic locations such as the “World Championship” or the “European Championship” had taken place in different locations over the years, so we did a mapping of logical to physical locations). Each race record corresponds to one triathlete´s participation in one race and includes the gender, age group, country of origin, race location, year of the race, etc. The number of records is the number of qualifying race results after discarding any records with bad or incomplete data. Race records are classified in 5-years age groups: 18–24, 25–29, 30–34, 35–39, 40–44, 45–49, 50–54, 55–59, 60–64, 65–69, 70–74, 75–79, 80–84 and 85–89 years following the general classification of age group triathletes in all triathlon races (www.triathlon.org/agegroup).

### Data analysis

Descriptive information is presented using frequencies, mean, standard deviation, and minimum and maximum values. A XG Boost Regression model was designed to use the race finish time (*FinishTime*) as the predicted variable, whereas *Gender*, *AgeGroup*, *Country EventLocation*, and the *Air* and *Water* temperatures were used as predictors. XG Boost stands for Extreme Gradient Boosting and is an optimized decision tree-based ensemble algorithm that uses gradient boosting and parallelization to outperform other algorithms in execution speed and prediction accuracy (www.sciencedirect.com/topics/computer-science/extreme-gradient-boosting). Whilst the air and water temperature variables were originally numerical, the other four predictive variables were of a categorical type and had to be numerically encoded before they could be used to train the model. The variable *Gender* was encoded as 0 (females) or 1 (males). The *AgeGroup* variable was encoded using the first two digits of the age group (so 18–24 became 18, 25–29 became 25, etc.). The *Country* variable was ranked by participation, that is, by number of race records, and the resulting order was used as the encoding index (so US is index 0 with the largest number of records.) Last, the *EventLocation* variable was ranked by average race finish time, with the fastest locations first. The dataset was then split randomly into two subsets, and the model was trained with 75% of the available data and validated against the remaining 25%. Two evaluation metrics were calculated: the Mean Absolute Error (MAE) and R-squared (coefficient of determination or R^2^). Several models were built to explore the best hyper-parameters combination. The optimal model was finally built with 1000 estimators (decision trees) with max depth of 5, and was trained with a learning rate of 0.2. All other model hyper-parameters were left as default. Model generalization (*i*.*e*. the ability of a predictive model to make accurate prediction with previously unseen data examples) was not among our priorities, as we rather sought to use the model with statistical descriptive purposes. After training the model, we asked what it learnt from the training data in order to understand how each feature contributes to the model predictions. Several techniques were used to this end: the model SHAP values and features relative importances, and the PDP-based model interpretation charts. SHAP values (SHapley Additive exPlanations) are a model-agnostic technique with origins in the cooperative game theory, which is widely used to increase the transparency and interpretability of machine learning models. SHAP values indicate both a measure of the importance of each feature and whether each feature has a positive or negative impact on the predictions. These impacts can be calculated for each individual prediction, for the whole validation data sample (in their aggregated form), or even for the combined effects of two features. The Partial Dependence Plots (PDP) model interpretability charts add an additional level of detail, showing the model distribution of race time predictions against reference averages and group sizes. The predictions distributions are represented by means of boxplots. All analyses, modeling and visualizations were done using Python (www.python.org/) and the specific statistical and machine learning libraries using a Google Colab notebook (https://colab.research.google.com/). The main libraries used in the study are: numpy, pandas, scipy, statsmodels, matplotlib, seaborn, country_converter, sklearn, xgboost, pdpbox, shap.

## Results

Most of the finishers were competing in IM triathlons held in the USA such as IM Wisconsin, IM Florida, IM Lake Placid, IM Arizona, or IM Hawaii, where the IM World Championship took place (**Table 1**). The fastest average overall race times were achieved in IM Hawaii, IM Austria, IM Copenhagen, IM Brazil Florianopolis, and IM Barcelona (**Table 2)**.

**Table 1:** Full list of the 66 IM race locations ranked by participation (number of finishers records).

	Event Location	Number of Races	Number of Records	Number of Unique Athletes
**0**	IRONMAN^®^ Wisconsin	19	38,545	26,986
**1**	IRONMAN^®^ Florida	19	38,157	29,081
**2**	IRONMAN^®^ Lake Placid	17	34,341	24,382
**3**	IRONMAN^®^ Arizona	17	34,246	25,689
**4**	IRONMAN^®^ Hawaii	18	32,156	21,080
**5**	IRONMAN^®^ Austria	16	30,970	23,560
**6**	IRONMAN^®^ France	16	29,302	24,200
**7**	IRONMAN^®^ Canada	15	27,135	19,771
**8**	IRONMAN^®^ Coeur d’Alene	15	24,526	19,575
**9**	IRONMAN^®^ Louisville	10	21,661	17,907
**10**	IRONMAN^®^ Texas	11	21,137	16,001
**11**	IRONMAN^®^ Frankfurt	13	20,057	16,579
**12**	IRONMAN^®^ Cozumel	13	17,902	14,470
**13**	IRONMAN^®^ Copenhagen	8	16,739	13,344
**14**	IRONMAN^®^ New Zealand	18	16,623	11,218
**15**	IRONMAN^®^ Mont-Tremblant	8	16,306	12,951
**16**	IRONMAN^®^ UK	11	15,221	12,066
**17**	IRONMAN^®^ Kalmar	8	14,640	9,826
**18**	IRONMAN^®^ Lanzarote	11	14,530	11,066
**19**	IRONMAN^®^ Brazil Florianopolis	10	14,010	9,656
**20**	IRONMAN^®^ Australia ‐ New South Wales	12	13,668	8,454
**21**	IRONMAN^®^ Zurich Switzerland	9	13,199	11,502
**22**	IRONMAN^®^ Wales	8	12,496	9,157
**23**	IRONMAN^®^ Barcelona	6	11,137	10,132
**24**	IRONMAN^®^ Chattanooga	6	10,788	9,605
**25**	IRONMAN^®^ Western Australia	10	10,651	7,835
**26**	IRONMAN^®^ South Africa	7	10,407	7,132
**27**	IRONMAN^®^ Boulder	6	8,492	7,244
**28**	IRONMAN^®^ Maryland	6	8,391	7,448
**29**	IRONMAN^®^ Cairns	10	8,253	6,348
**30**	IRONMAN^®^ Emilia Romagna	4	8,065	7,261
**31**	IRONMAN^®^ Vichy	6	7,419	6,798
**32**	IRONMAN^®^ Hamburg	4	6,654	6,249
**33**	IRONMAN^®^ Mallorca	5	6,262	5,790
**34**	IRONMAN^®^ Malaysia	8	5,409	4,255
**35**	IRONMAN^®^ Melbourne	4	4,667	3,985
**36**	IRONMAN^®^ Santa Rosa	3	4,655	4,275
**37**	IRONMAN^®^ Maastricht	4	4,066	3,737
**38**	IRONMAN^®^ St. George	3	3,705	3,394
**39**	IRONMAN^®^ Japan	3	3,515	2,746
**40**	IRONMAN^®^ Tallinn	4	3,333	3,009
**41**	IRONMAN^®^ Regensburg	2	2,825	2,703
**42**	IRONMAN^®^ Los Cabos	5	2,645	2,404
**43**	IRONMAN^®^ Gurye Korea	2	2,609	2,251
**44**	IRONMAN^®^ Lake Tahoe	2	2,605	2,504
**45**	IRONMAN^®^ Tulsa	2	2,311	2,244
**46**	IRONMAN^®^ New York	1	1,955	1,950
**47**	IRONMAN^®^ Taiwan	4	1,940	1,739
**48**	IRONMAN^®^ Brazil Fortaleza	3	1,755	1,484
**49**	IRONMAN^®^ Vineman	1	1,699	1,696
**50**	IRONMAN^®^ Vitoria-Gasteiz	1	1,572	1,568
**51**	IRONMAN^®^ Mar del Plata	3	1,492	1,372
**52**	IRONMAN^®^ Portugal-Cascais	1	1,390	1,384
**53**	IRONMAN^®^ Subic Bay Philippines	2	1,262	1,172
**54**	IRONMAN^®^ Indiana	1	1,223	1,223
**55**	IRONMAN^®^ Muskoka	1	1,046	1,044
**56**	IRONMAN^®^ Switzerland Thun	1	884	884
**57**	IRONMAN^®^ Des Moines	1	843	842
**58**	IRONMAN^®^ Haugesund Norway	2	825	779
**59**	IRONMAN^®^ Weymouth	1	700	697
**60**	IRONMAN^®^ Waco	1	639	639
**61**	IRONMAN^®^ Finland Kuopio-Tahko	1	624	624
**62**	IRONMAN^®^ Gdynia	1	590	588
**63**	IRONMAN^®^ China	2	415	385
**64**	IRONMAN^®^ Pays D’Aix	1	383	383
**65**	IRONMAN^®^ North Carolina	1	34	34

**Table 2:** Top 25 IM race locations by average finish time. Times are presented as hours:minutes:seconds.

	Event Location	Race finish time				
		Records	mean	std	min	max
**0**	IRONMAN^®^ Vitoria-Gasteiz	1,572	11:35:17	01:21:41	08:46:18	15:42:38
**1**	IRONMAN^®^ Copenhagen	16,739	11:41:04	01:22:43	08:19:14	16:06:57
**2**	IRONMAN^®^ Hawaii	32,156	11:43:02	01:51:33	08:24:36	17:50:33
**3**	IRONMAN^®^ Tallinn	3,333	11:43:59	01:36:07	08:27:45	16:59:37
**4**	IRONMAN^®^ Regensburg	2,825	11:45:31	01:28:43	08:42:34	15:55:22
**5**	IRONMAN^®^ Barcelona	11,137	11:46:41	01:25:56	08:22:24	16:01:29
**6**	IRONMAN^®^ Brazil Florianopolis	14,010	11:48:13	01:31:03	08:36:29	17:32:34
**7**	IRONMAN^®^ Hamburg	6,654	11:50:55	01:30:02	08:12:43	15:48:47
**8**	IRONMAN^®^ Mallorca	6,262	11:54:14	01:34:26	08:16:48	16:23:56
**9**	IRONMAN^®^ Austria	30,970	11:55:11	01:35:13	08:33:27	17:10:50
**10**	IRONMAN^®^ Switzerland Thun	884	11:55:40	01:33:31	08:28:57	16:27:00
**11**	IRONMAN^®^ Emilia Romagna	8,065	11:57:27	01:33:22	08:34:42	15:58:53
**12**	IRONMAN^®^ Finland Kuopio-Tahko	624	11:58:28	01:25:19	09:09:01	15:49:35
**13**	IRONMAN^®^ Melbourne	4,667	12:00:33	01:41:59	08:47:42	17:03:49
**14**	IRONMAN^®^ Frankfurt	20,057	12:01:36	01:23:00	08:43:51	15:53:45
**15**	IRONMAN^®^ Kalmar	14,640	12:04:54	01:27:55	08:29:15	16:15:18
**16**	IRONMAN^®^ Maastricht	4,066	12:09:22	01:32:00	08:55:44	16:40:13
**17**	IRONMAN^®^ Mar del Plata	1,492	12:09:40	01:36:57	08:46:41	16:54:51
**18**	IRONMAN^®^ Gdynia	590	12:17:43	01:33:46	08:52:37	16:19:51
**19**	IRONMAN^®^ Portugal-Cascais	1,390	12:18:33	01:34:26	08:56:28	15:57:07
**20**	IRONMAN^®^ Vichy	7,419	12:18:59	01:37:07	08:23:49	16:55:55
**21**	IRONMAN^®^ Pays D’Aix	383	12:20:15	01:34:31	08:47:05	15:58:45
**22**	IRONMAN^®^ Zurich Switzerland	13,199	12:21:28	01:31:56	08:52:38	16:01:34
**23**	IRONMAN^®^ Haugesund Norway	825	12:22:24	01:32:27	08:48:41	16:15:48
**24**	IRONMAN^®^ France	29,302	12:23:18	01:31:59	08:20:08	16:50:25

Regarding the triathlete´s countries of origin, the vast majority of the successful finishers originated from the US, followed by athletes from the UK, Canada, Australia, Germany, and France (**Table 3**), but the best mean times were achieved by athletes from Austria, Germany, Belgium, Switzerland, Finland, and Denmark.

**Table 3:** Ranking of top 25 countries by number of records of IM finishers, accounting for over 94% of the full sample. Times are presented as hours:minutes:seconds.

				Overall race time			
	Country	Number of Records	Number of Unique Athletes	mean	std	min	max
**0**	USA	27,4553	12,4646	13:21:38	01:45:05	08:17:24	17:57:34
**1**	GBR	55,410	29,852	13:02:11	01:39:05	08:31:52	17:04:40
**2**	CAN	38,264	18,074	13:01:58	01:43:45	08:23:48	17:50:43
**3**	AUS	37,571	16,836	12:15:32	01:40:10	08:39:10	17:03:49
**4**	DEU	32,662	16,240	11:44:13	01:30:45	08:12:43	17:35:15
**5**	FRA	27,873	16,293	12:06:37	01:32:35	08:19:20	17:02:02
**6**	ESP	17,009	9,788	12:04:09	01:35:02	08:06:57	16:57:55
**7**	SWE	14,640	7,057	12:05:09	01:28:52	08:30:55	17:50:33
**8**	BRA	14,408	7,911	11:51:22	01:35:39	08:29:48	17:32:34
**9**	AUT	12,792	6,267	11:40:39	01:29:42	08:37:13	16:56:30
**10**	ITA	12,702	6,236	12:05:08	01:28:46	08:35:54	16:50:26
**11**	MEX	11,941	6,211	13:13:53	01:38:57	08:36:19	17:05:38
**12**	DNK	11,874	6,469	11:35:16	01:23:42	08:22:31	17:07:48
**13**	NZL	11,092	5,538	12:41:41	01:45:16	08:03:41	17:38:06
**14**	JPN	10,249	4,990	13:27:16	01:49:16	08:20:05	17:00:31
**15**	ZAF	9,675	5,732	13:17:24	01:38:51	08:41:51	16:58:05
**16**	BEL	8,444	3,896	11:28:32	01:28:10	08:16:08	16:59:56
**17**	CHE	7,570	3,381	11:37:15	01:29:16	08:23:49	16:53:40
**18**	IRL	6,337	3,625	12:32:17	01:39:02	08:26:02	17:02:23
**19**	ARG	5,470	2,679	12:06:03	01:35:32	08:33:44	17:19:28
**20**	NLD	4,750	2,841	11:49:39	01:30:01	08:16:48	16:59:30
**21**	FIN	3,497	1,785	11:40:55	01:23:43	08:32:40	16:35:12
**22**	POL	3,438	2,005	12:00:30	01:31:23	08:46:49	16:44:55
**23**	RUS	3,163	1,953	11:54:43	01:30:19	08:22:24	17:03:00
**24**	ISR	2,952	1,884	12:44:26	01:35:25	08:40:21	16:53:06

**Table 4** presents the percentage of unsuccessful finishers (DNF) in all IM races sorted in alphabetical order of the races. The highest percentage of non-finishers was recorded for IM Korea, followed by IM North Carolina and IM Taiwan.

**Table 4:** The percentage of non-finishers (DNF) for each IM race sorted in alphabetical order.

	Event Status			
EventLocation	DNF	Finish	Both	DNF rate
IRONMAN^®^ Arizona	68	34325	34393	0,20%
IRONMAN^®^ Australia ‐ New South Wales	11	13673	13684	0,08%
IRONMAN^®^ Austria	1	33261	33262	0,00%
IRONMAN^®^ Barcelona	2	11141	11143	0,02%
IRONMAN^®^ Boulder	38	8494	8532	0,45%
IRONMAN^®^ Brazil Florianópolis	10	14021	14031	0,07%
IRONMAN^®^ Brazil Fortaleza	4	1756	1760	0,23%
IRONMAN^®^ Cairns	49	9458	9507	0,52%
IRONMAN^®^ Canada	26	27146	27172	0,10%
IRONMAN^®^ Chattanooga	78	10799	10877	0,72%
IRONMAN^®^ China	0	415	415	0,00%
IRONMAN^®^ Coeur d’Alene	31	24542	24573	0,13%
IRONMAN^®^ Copenhagen	24	16751	16775	0,14%
IRONMAN^®^ Cozumel	87	17933	18020	0,48%
IRONMAN^®^ Des Moines	14	843	857	1,63%
IRONMAN^®^ Emilia Romagna	11	8078	8089	0,14%
IRONMAN^®^ Finland Kuopio-Tahko	0	624	624	0,00%
IRONMAN^®^ Florida	134	38204	38338	0,35%
IRONMAN^®^ France	7	29337	29344	0,02%
IRONMAN^®^ Frankfurt	1	23342	23343	0,00%
IRONMAN^®^ Gdynia	0	601	601	0,00%
IRONMAN^®^ Gurye Korea	81	2609	2690	3,01%
IRONMAN^®^ Hamburg	23	6668	6691	0,34%
IRONMAN^®^ Haugesund Norway	1	825	826	0,12%
IRONMAN^®^ Hawaii	40	32186	32226	0,12%
IRONMAN^®^ Indiana	17	1223	1240	1,37%
IRONMAN^®^ Japan	4	3516	3520	0,11%
IRONMAN^®^ Kalmar	0	14651	14651	0,00%
IRONMAN^®^ Lake Placid	93	34369	34462	0,27%
IRONMAN^®^ Lake Tahoe	2	2606	2608	0,08%
IRONMAN^®^ Lanzarote	5	14800	14805	0,03%
IRONMAN^®^ Los Cabos	0	2645	2645	0,00%
IRONMAN^®^ Louisville	50	21678	21728	0,23%
IRONMAN^®^ Maastricht	0	4068	4068	0,00%
IRONMAN^®^ Malaysia	79	5427	5506	1,43%
IRONMAN^®^ Mallorca	0	6265	6265	0,00%
IRONMAN^®^ Mar del Plata	2	1496	1498	0,13%
IRONMAN^®^ Maryland	10	8393	8403	0,12%
IRONMAN^®^ Melbourne	3	4669	4672	0,06%
IRONMAN^®^ Mont-Tremblant	21	16309	16330	0,13%
IRONMAN^®^ Muskoka	0	1047	1047	0,00%
IRONMAN^®^ New York	0	1955	1955	0,00%
IRONMAN^®^ New Zealand	10	16637	16647	0,06%
IRONMAN^®^ North Carolina	1	34	35	2,86%
IRONMAN^®^ Pays D’Aix	0	383	383	0,00%
IRONMAN^®^ Portugal-Cascais	11	1390	1401	0,79%
IRONMAN^®^ Regensburg	0	2826	2826	0,00%
IRONMAN^®^ Santa Rosa	19	4670	4689	0,41%
IRONMAN^®^ South Africa	24	11997	12021	0,20%
IRONMAN^®^ St. George	0	3708	3708	0,00%
IRONMAN^®^ Subic Bay Philippines	15	1262	1277	1,17%
IRONMAN^®^ Switzerland Thun	1	885	886	0,11%
IRONMAN^®^ Taiwan	49	2718	2767	1,77%
IRONMAN^®^ Tallinn	0	3333	3333	0,00%
IRONMAN^®^ Texas	82	21148	21230	0,39%
IRONMAN^®^ Tulsa	11	2312	2323	0,47%
IRONMAN^®^ UK	7	15224	15231	0,05%
IRONMAN^®^ Vichy	1	7457	7458	0,01%
IRONMAN^®^ Vineman	4	1699	1703	0,23%
IRONMAN^®^ Vitoria-Gasteiz	1	1572	1573	0,06%
IRONMAN^®^ Waco	3	639	642	0,47%
IRONMAN^®^ Wales	0	12505	12505	0,00%
IRONMAN^®^ Western Australia	28	10660	10688	0,26%
IRONMAN^®^ Weymouth	0	700	700	0,00%
IRONMAN^®^ Wisconsin	33	38568	38601	0,09%
IRONMAN^®^ Zurich Switzerland	7	13220	13227	0,05%
	1334	687696	689030	

Regarding the predictive model performance, the XG Boost model produced an MAE of 4503.6 seconds (approx. 1h15min) over the 25% test sample. This metric, however, does not tell us much about the “goodness of fit”, that is, whether the model has strong or weak predictive power. The R^2^ score is a better metric to get an idea of the algorithm’s accuracy. The model produced an R^2^ score of 0.27, meaning that 27% of the predicted race finish time variability can be explained through the joint effect of the six predicting variables. R^2^-values of up to 0.25 or 0.3 are usually considered weak effects. Naturally, this model is just too simplistic to accurately represent reality, and the R^2^ result just indicates that there must be other variables with a stronger influence on an IM race finish time. But at 0.27, it is not irrelevant, and we can still use several model interpretability tools to extract some insights as to how each feature contributed to the model predictions.

### Features relative importance (SHAP based)

**Fig 1:**
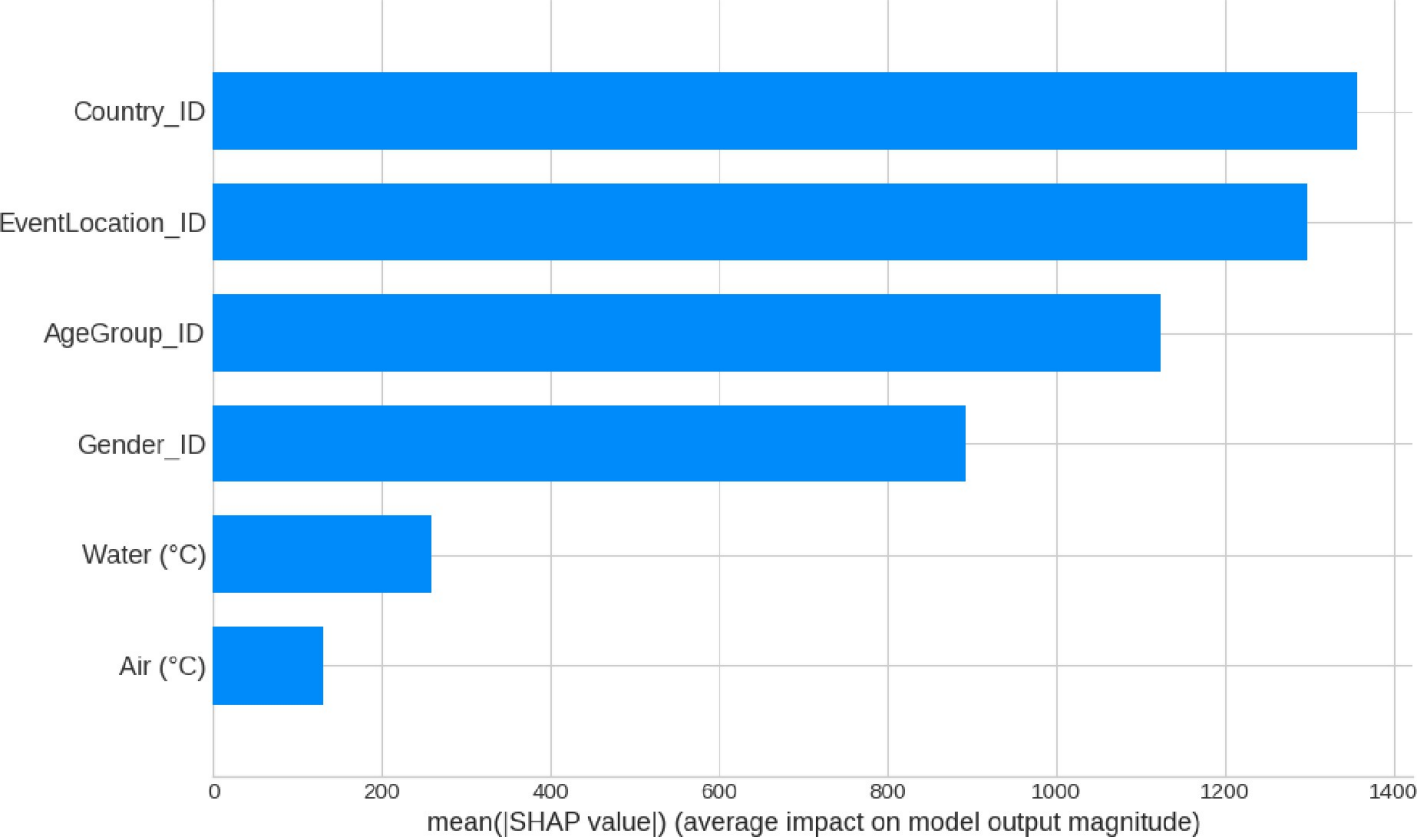
XG Boost Regressor features importances (SHAP-based).

Our XG Boost Regressor determined that *Country* (origin of the athlete) was the most important variable. However, the event location (where the race was held) wasthe feature with the second biggest relative importance (**Fig 1**).

### Aggregated SHAP values

**Fig 2:**
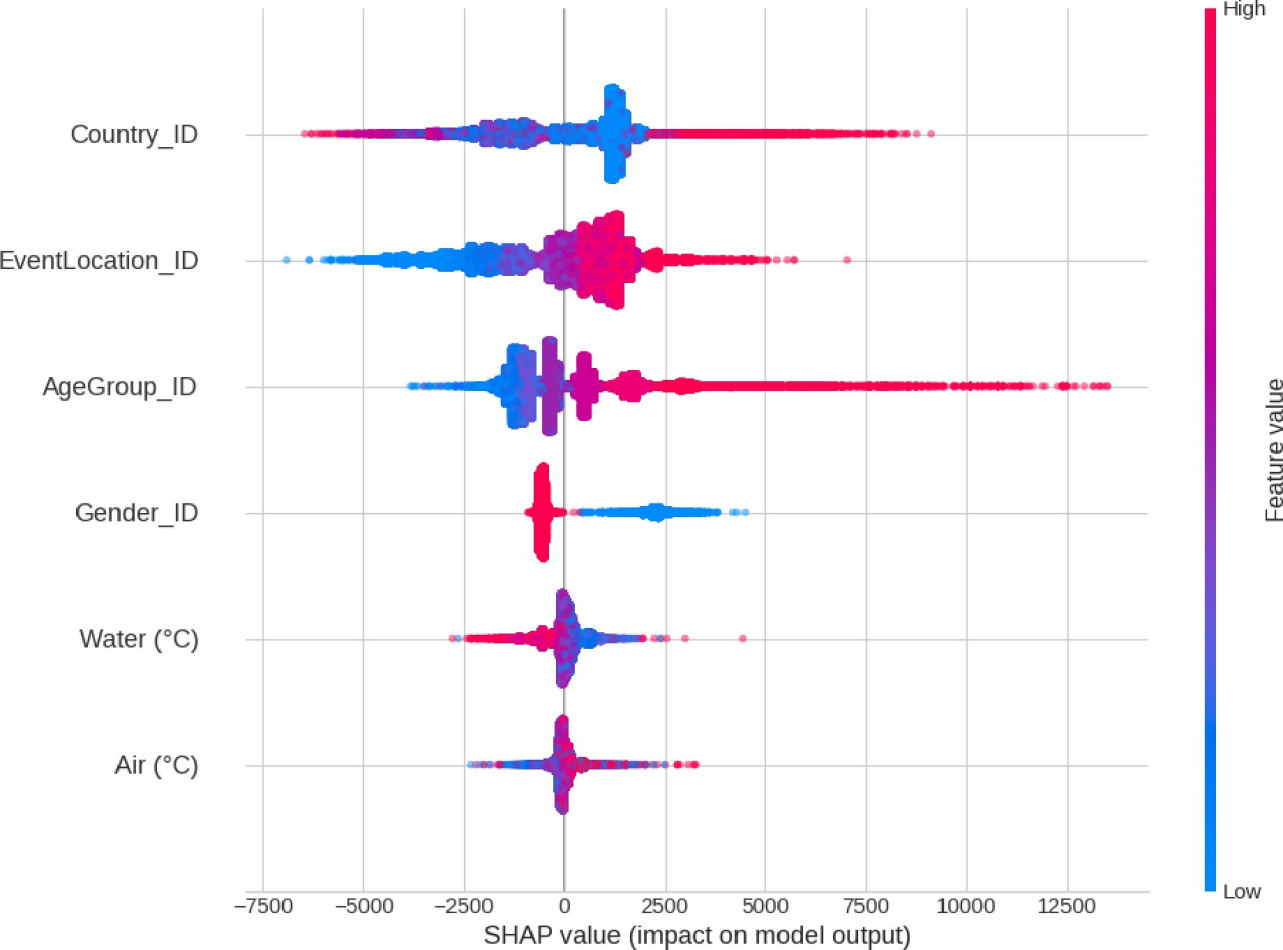
Aggregated SHAP values for the XG Boost predictive model.

**Fig 2** shows the aggregated SHAP values of the XG Boost model. SHAP values present a quantitative view of the amount of change in the output (predicted *FinishTime*) for each input (predictor). The x-axis (SHAP value) represents seconds added to (or subtracted from) the reference line (set at zero). For the *Country*, the blue points concentrated in several clusters around zero and left and right of the reference line. Blue points correspond to low values of *Country*_ID. These countries have the largest number of participants, which are the USA (by a large margin) followed by the UK, Canada, Australia, Germany, France, Spain, Sweden, Brazil, etc., indicating a broad distribution of performances at all levels of competence. Red dots represent countries with marginal participation and accumulate on the left and right extremes of the chart. The *EventLocation* feature produces a chart where blue dots (low values of event location) accumulate on the left side, whilst increasing values of the location ID (violets and reds) accumulate towards the right. Given the rank encoding based on the average race finish time, blue points represent the fastest IM event locations (IM Hawaii, IM Copenhagen, IM Tallinn, IM Vitoria-Gasteiz, IM Regensburg, IM Barcelona, and IM Brazil Florianopolis. The *AgeGroup* feature gives the broadest range of change in the output (from around -3,000 to well over +12,000 seconds, about 4 hours). The red dots represent the older age groups making positive contributions to the *FinishTime* predictions whilst blue dots (low age groups, i.e., 18, 25, 30…) cluster on the negative side of the chart, confirming low age as a decisive predicting factor in deducting seconds from the race time. And in the last place, the *Gender* feature contributes to the prediction in an almost binary way, either adding seconds for females (value 0) or subtracting for males (value 1).

### Advanced model interpretability charts

**Fig 3:**
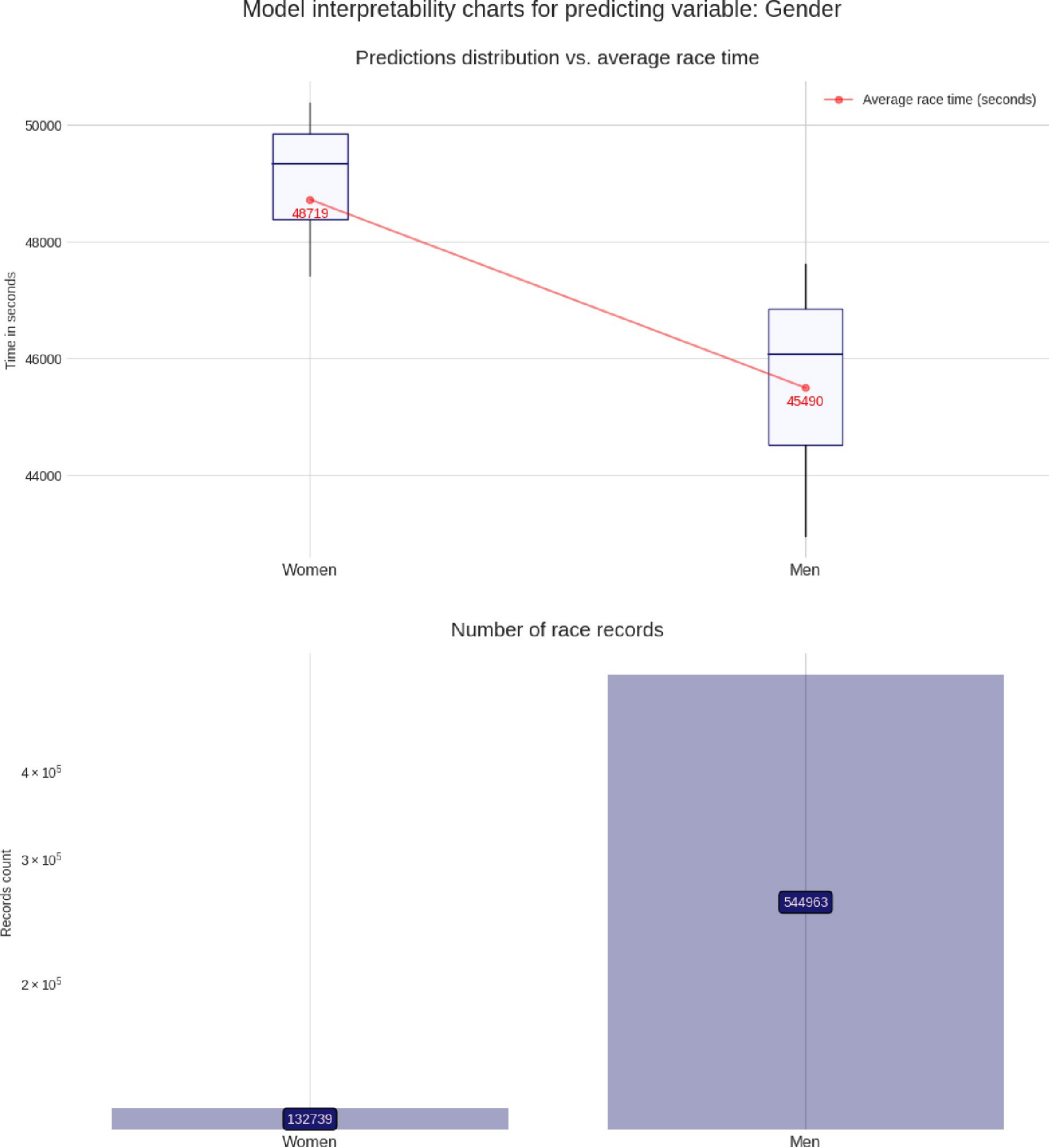
Advanced model interpretability charts for gender.

**Fig 4:**
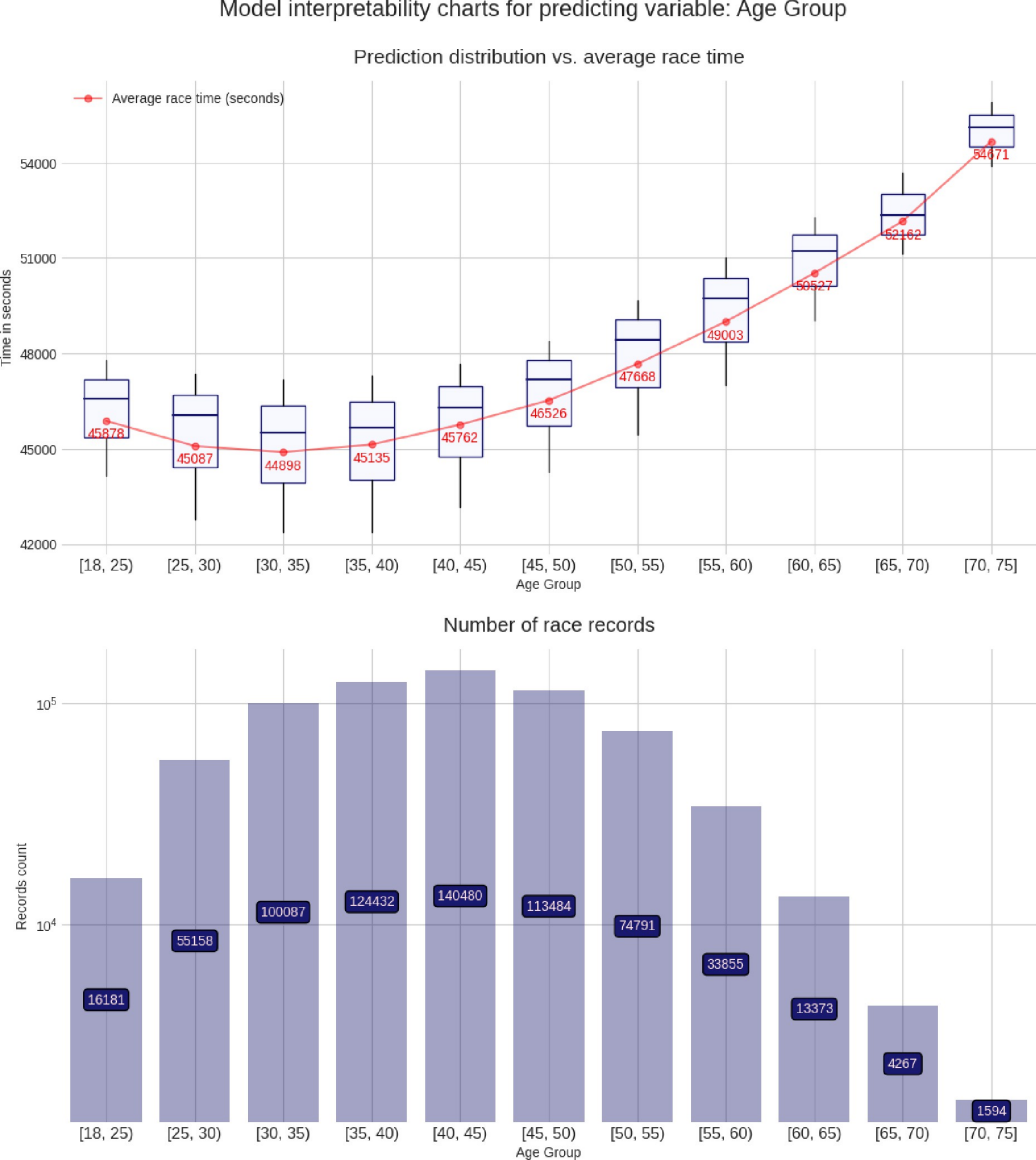
Advanced model interpretability charts for age group.

**Fig 5:**
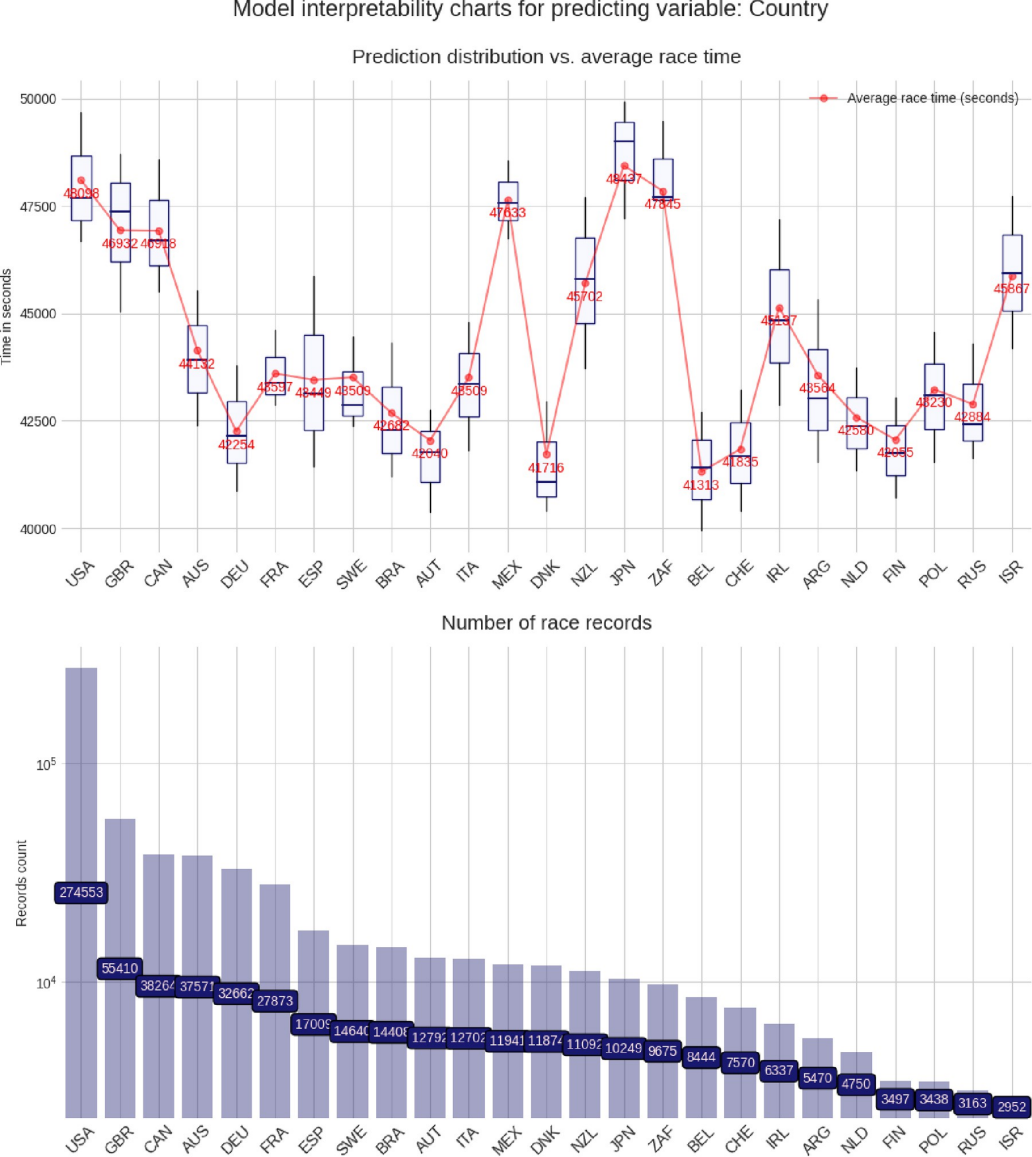
Advanced model interpretability charts for country of origin of the athlete.

**Fig 6:**
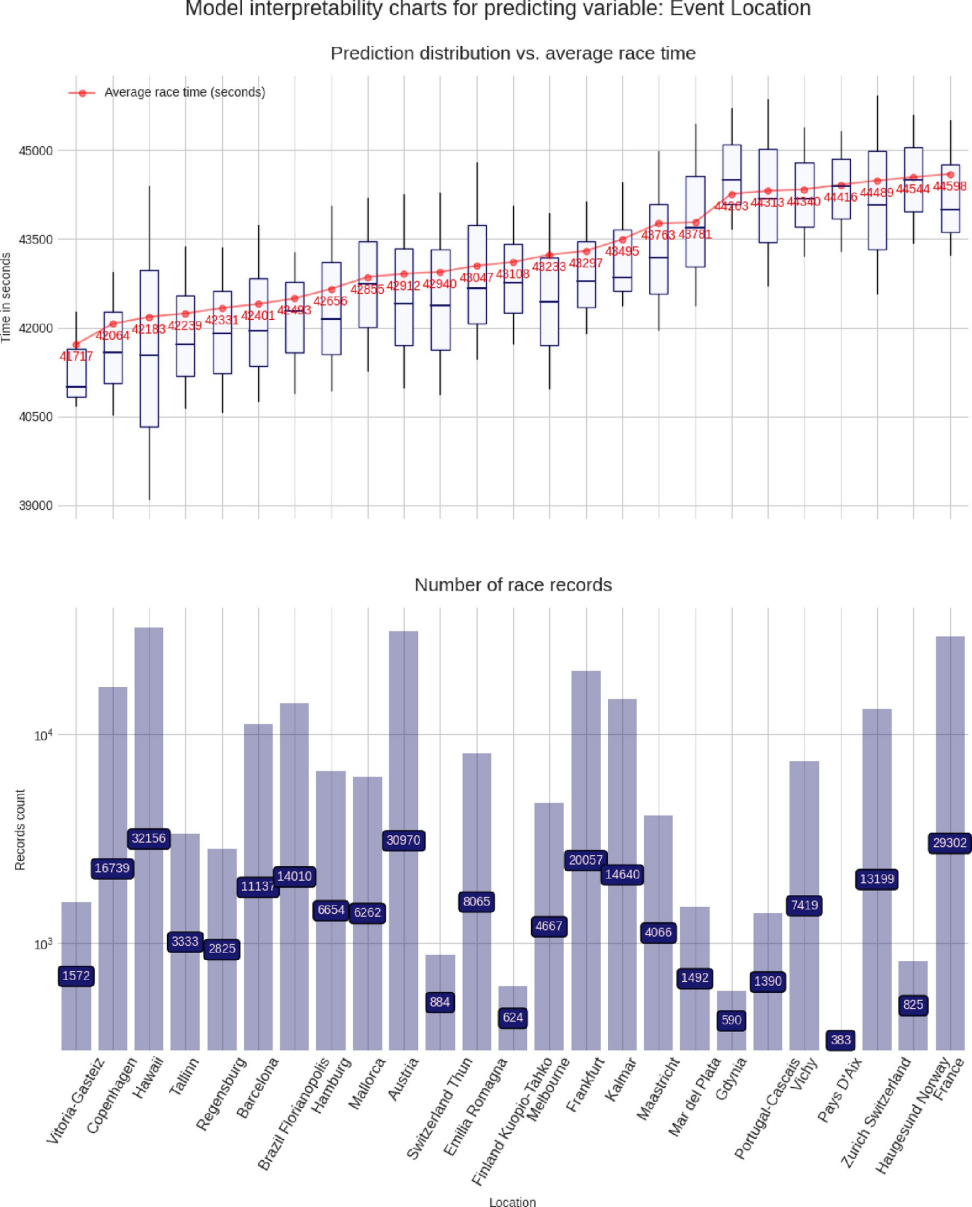
Advanced model interpretability charts for country of event location.

**Fig 7:**
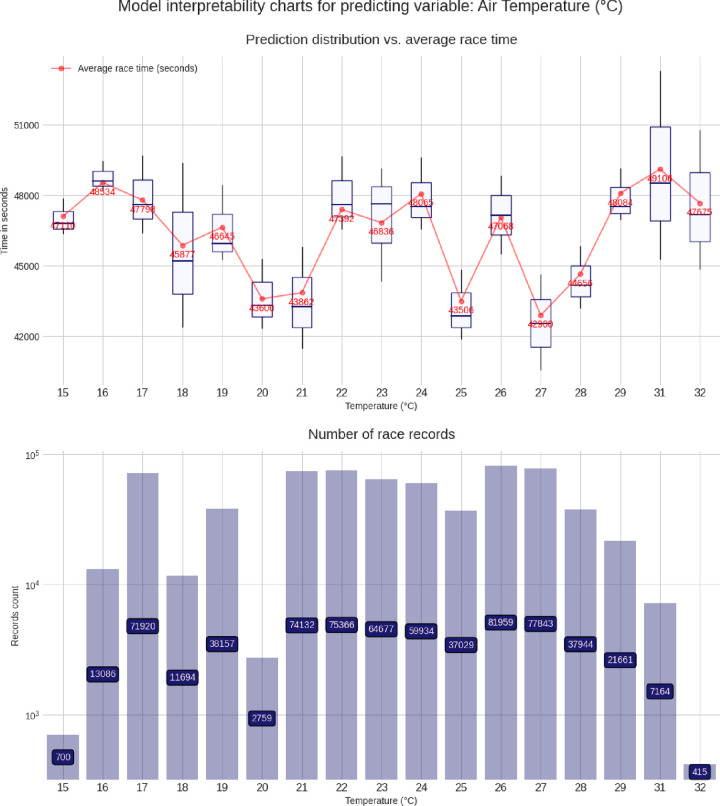
Advanced model interpretability charts for air temperature.

**Fig 8:**
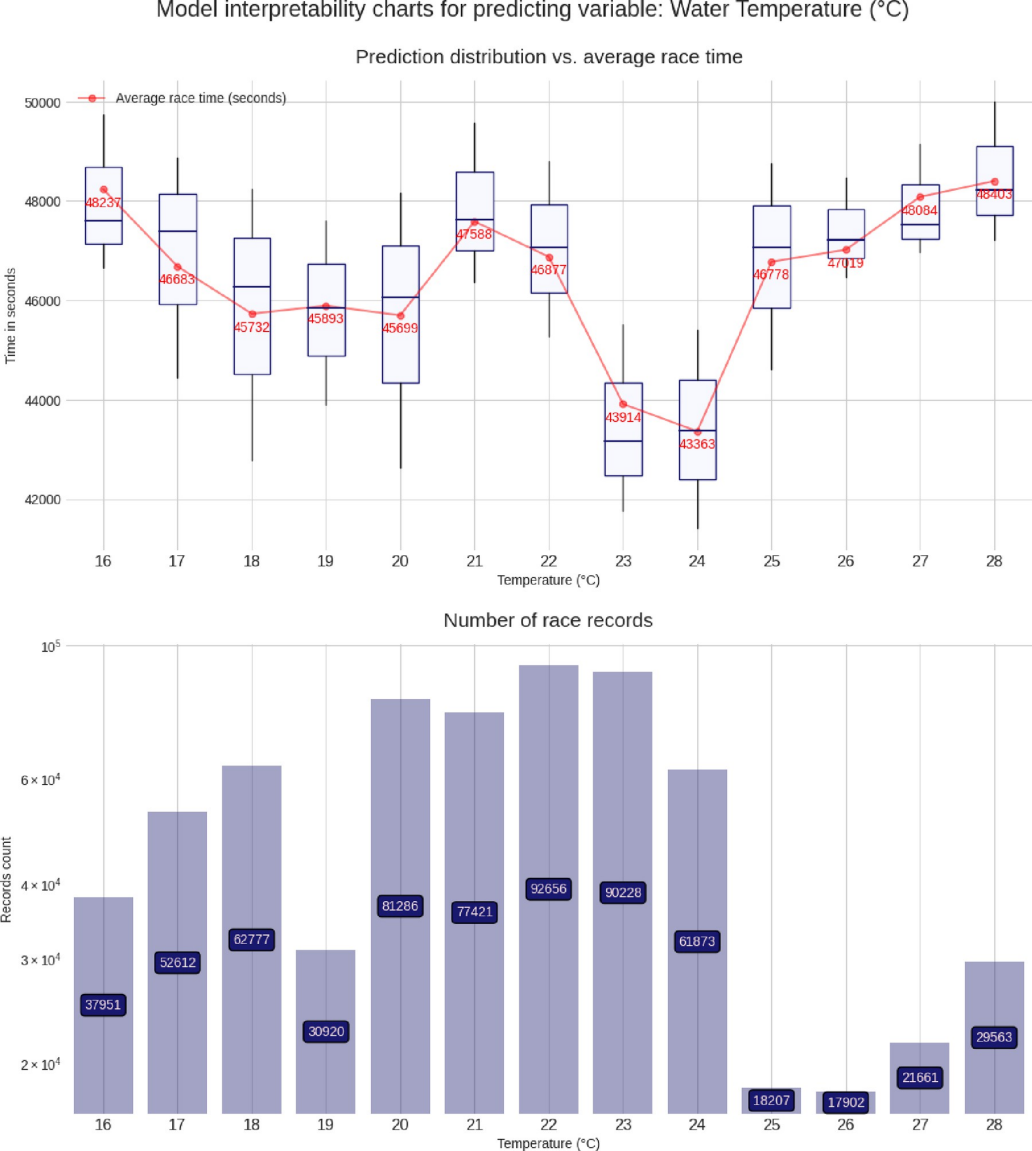
Advanced model interpretability charts for water temperature.

These charts are built using the pdpbox and matplotlib libraries, to produce a customized view of our model. For each predicting variable (gender, age group, air temperature, etc.), a set of two charts is produced, describing the relationship between that predicting variable and the target variable (*i*.*e*. the race time in seconds). The lower chart is just a bar chart specifying the groups sizes (for instance, for the gender chart group, it represents the number of race records from male athletes and from female athletes). The upper chart is the most interesting and represents the model prediction distribution, for each possible value of the predicting variable visualized as box plots, i.e through the min, max, 25%, 50% and, 75% percentile values, and against the average group value in red. More men than women competed and men were faster than women (**Fig 3**). Athletes in age group 40–45 years were the most numerous, but athletes in age group 30–35 years were the fastest (**Fig 4**). Athletes from the USA were the most numerous, but athletes from Belgium, Denmark, Austria, Switzerland, Germany, and Finland were the fastest (**Fig 5**). The fastest overall race times were achieved in IM Hawaii (**Fig 6**). Regarding environmental conditions, the best race times were obtained an air temperature of ∼27°C (**Fig 7**) and a water temperature of ∼24°C (**Fig 8**).

## Discussion

This study intended to find the fastest racecourse for age group triathletes competing in IM triathlons by interpreting an XG Boost predictive model. Based on existing literature, we hypothesized that most IM age group triathletes would originate from the US and they would preferably compete in IM races held in the US. We found that (i) IM Hawaii was the fastest racecourse overall, (ii) most of the successful and most numerous IM finishers originated from the US, but athletes from Belgium, Denmark, Austria, Switzerland, Germany, and Finland were the fastest, (iii) athletes in age group 40–45 were the most numerous, but athletes in age group 30–35 years were the fastest and (iv) the best IM race times were achieved at an air temperature of ∼27°C and a water temperature of ∼24°C.

### IM Hawaii is the fastest racecourse for IM age group triathletes

The first and most important finding was that the racecourse of IM Hawaii was the fastest course for most IM age group triathletes. It is well known that IM age group triathletes intending to compete in the IM World Championship IM Hawaii need to qualify [8]. For example, for 2023, IM age group triathletes have eight possibilities to qualify for the IM World Championship (www.ironman.com/news_article/show/1241591). The standard qualification competes in one of the 47 full-distance IM races in the qualifying series. A specific number of qualification slots are offered for each age group regarding the number of finishers per age group (www.ironman.com/im-world-championship-2023-slot). These characteristics guarantee the best IM athletes competing in this event, given that sustained performance and a high level of performance throughout the season are required. This qualification system works since only the best qualifiers can compete in IM Hawaii. Future studies need to consider mixed methods to understand the atmosphere behind this event, including motivational aspects and preparation characteristics.

### US-American IM triathletes were the most numerous but not the fastest

A second important finding was that most of the successful IM finishers originated from the USA, followed by athletes from the UK, Canada, Australia, Germany, and France, but athletes from Belgium, Denmark, Austria, Switzerland, Germany, and Finland achieved the best mean times. These findings confirmed our first hypothesis that most IM age group triathletes would originate from the USA, but IM age group triathletes from the USA were not the fastest.

To date, three studies investigated the aspect of nationality and performance in an IM triathlon. One study analyzed participation and performance in the IM World Championship IM Hawaii and its qualifying races in 2010 showed that IM triathletes from the USA were both the most numerous and the fastest [8]. A study investigating a larger sample of finishers of IM Hawaii over a longer time frame confirmed that successful finishers at IM Hawaii were mainly US-American triathletes [8]. A study analyzing more than 300,000 IM triathletes showed that US-Americans were not the fastest at IM Hawaii, but IM triathletes originating from Germany, Australia, Austria, and Brazil [10]. In the present analysis with more than 670,000 finishers, IM triathletes from Austria, Germany, Belgium, Switzerland, Finland, and Denmark were the fastest, confirming recent findings investigating half of the sample and during an earlier time frame.

The finding that local athletes are not the fastest has already been shown in open-water long-distance swimmers. For example, in the ‘English Channel Swim’, where open-water swimmers cross the ‘English Channel’ between England and France, most swimmers were from Great Britain, USA, Australia and Ireland. However, swimmers from the USA and Australia achieved the fastest swim times before swimmers from Great Britain [20]. However, in crossing the ‘Strait of Gibraltar’, local Spanish swimmers were faster than US-American swimmers [21].

It is also important to note that Hawaii is within the territory of the USA but quite far from the mainland. For example, the time difference between New York and Hawaii is 6 hours. Furthermore, Hawaii has a tropical climate. A different time zone and a different climate might counteract the advantages of locals (i.e., US-Americans). Similar to data about the fastest racecourse, future studies need to consider different research approaches in order to expand our understanding of how the travel distance, the knowledge about the racecourse, and geographical characteristics affect athletes’ participation and performance.

### Most of the finishers were competing in IM triathlons held in the USA

We could confirm our second hypothesis that most IM age group triathletes would compete in IM races held in the USA. Most of the successful finishers were competing in IM triathlons held in the USA, such as IM Wisconsin, IM Florida, IM Lake Placid, IM Arizona, and IM Hawaii, where the IM World Championship took place.

The fact that local athletes mainly compete in their home country has been described in different races such as the ‘Swiss Alpine Marathon’ in Switzerland where Swiss athletes were the most numerous and the best [22]. Similarly, in the ‘Powerman Duathlon World Championship’ held in Switzerland, duathletes from Switzerland were the most numerous and the fastest [23]. Also, Swiss triathletes represented the largest group of participants and were the fastest finishers in IM Switzerland held in Zurich, Switzerland [24].

An explanation of the increased number of local athletes in these races might be the advantages of competing at home in terms of budget, traveling and time management, as well as adaptation to weather conditions and no need to adapt to time zones. Furthermore, it should be noted that the above-mentioned races occur in countries where multi-sports are popular, which facilitates increased rates of participation of local athletes. The host effect was presented previously in the context of the Olympic Games [25]. Positive effects were shown for political, economic, cultural, environmental, tourism, and psychological aspects, as well as the number of medals in hosting countries’ [25]. In another way, little information is available about the role of hosting events in triathlon performance. Future studies need to consider investigating the relationship between the number of events, countries’ performance, and racecourse characteristics.

### The fastest IM race times at the age of 30–35 years

A further important finding was that the fastest IM race times were achieved at the age of 30–35 years. The age of 35 years corresponds to the age of peak IM race performance, which has been described and confirmed in several studies investigating different samples of IM triathletes [6, 26, 27]. One study specifically investigated the age of peak triathlon performance in elite IM triathletes competing in IM Hawaii [28]. In the annual top ten women and men competing in the IM World Championship IM Hawaii between 1983 and 2012, the age of the annual top ten women and men increased over time to 35±5 and 34±3 years, respectively [28]. Lepers *et al*. suggested that the age-related decline in performance in the IM distance was larger than in the Olympic distance and attributed this observation to the longer duration of the former race [29].

The age of peak performance in the IM distance observed in the present study was older than what was shown by Malcata *et al*. in the Olympic distance triathlon [30], highlighting the role of the race distance. Although the purpose of this study was not to find the age of peak performance, our findings reinforce the interplay of different variables (*i*.*e*. sex, country, and event location) to the performance of amateur (*i*.*e*. recreational or age group) triathletes. Despite the fastest race time, as shown at the age of 35 years and younger, the IM Hawaii presented the higher contribution to athletes’ performance. However, the magnitude of this contribution was different for athletes of different ages. These initial results reinforce the complexity associated with endurance performance and suggest that future studies need to consider a holistic approach that considers the interplay between different variables and characteristics.

### The aspect of environmental conditions

A last important finding was that the best IM race times were obtained at an air temperature of ∼27°C and at a water temperature of ∼24°C. It is well known that environmental conditions can have an impact on ultra-endurance performance [31–33]. A very recent study investigating Olympic distance triathletes competing in China between 2015 and 2021 showed that the specific split disciplines cycling and running had a different impact on overall race time depending upon the environmental temperatures [19]. Regarding swimming in a full-distance triathlon such as the ‘Norseman Xtreme Triathlon’, a water temperature of 14.4–16.4°C may lead to hypothermia even when wearing a wetsuit [34]. On the other hand, running in IM Hawaii at 22.44–28.50°C, high-level age group triathletes reached a state of hyperthermia during the marathon [35]. Both conditions of hypothermia in swimming and hyperthermia in running might have a considerable impact on overall race time in an IM triathlon. Most probably the air temperature of ∼27°C and the water temperature of ∼24°C were optimal temperatures for a fast race time in IM Hawaii.

World Triathlon has established specific rules regarding environmental temperatures (www.triathlon.org/about/downloads/category/competition_rules). Specifically for high ambient temperatures, prevention guidelines have been established for triathlon races (https://www.triathlon.org/uploads/docs/World_Triathlon_Guidelines_for_Exertional_Heat_Illness_Prevention_201902261.pdf). Also for running races, specific rules have been set (IIRM_Brochure_AW_Lo.pdf (emos1storage.blob.core.windows.net). Overall, high temperatures should be avoided for endurance athletes. For example, it was suggested that road-based marathon running races should not be started when wet bulb globe temperature (WBGT) at the start is > 21°C [36]. Another study defined upper thresholds for marathon to WBGT 24–27°C and for triathlon to WBGT 23–26°C [37]. Interestingly, these IM triathletes were the best at higher ambient temperatures than suggested in literature.

The question remains now whether the environmental conditions have an influence on race performance or not. IM age group athletes who intend to qualify for the IM World Championship in IM Hawaii compete in different races [8, 9, 11] with different environmental conditions as described. Most probably the selection of the best IM age group athletes leads to the fact that only the most experienced and fastest IM age group athletes can compete in IM Hawaii also evidenced by the very low non-finisher race of 0.12%. We assume that the best IM age group athletes are not affected by environmental conditions.

### Limitations and implications for future studies

Although this analysis of a very large data set provides valuable insights for athletes and coaches regarding the selection of the fastest IM racecourse, some limitations should be acknowledged. The races provided only a general temperature for air and water temperatures and an overall description of the cycling and running course. Aspects such as food [3] and fluid [38] intake, hydration status [39] and thermoregulation [40], pacing during the race [41], elevations in the race course [41] and previous experience [2] were not considered. All these aspects might have a considerable influence on IM race performance. Future studies could benefit from using a qualitative approach to deeply understand the athletes’ perception of the racecourse characteristics and the motivation and budget associated with participating in events (i.e., training preparation and travel distances). Detailed information about weather (e.g. wind speed, humidity, solar radiation, altitude, and changes in elevation would be helpful for a detailed analysis. Future studies might incorporate detailed weather data from the specific regions using a high temporal resolution. Another important point is to deeply understand why low and middle-income countries are unrepresentative in participation and performance in IM race events. On the other hand, the study’s strength was its novelty, as it was the first to examine this topic using this methodological approach (the popular machine learning XG Boost algorithm). Our findings have practical information for triathletes in the context of selecting suitable races to compete in, especially considering the increased budget of participating and associated training workload.

## Conclusion

In summary, IM Hawaii was the fastest racecourse overall for IM age group triathletes, most of the successful IM finishers originated from the US, but athletes from Belgium, Denmark, Austria, Switzerland, Germany, and Finland were the fastest, athletes in age group 40–45 were the most numerous, but athletes in age group 30–35 years were the fastest and the best race times were obtained an air temperature of ∼27°C and a water temperature of ∼24°C. The results of the present study are helpful for coaches and athletes to attend the most important events and fastest racecourses.

## Supporting information

S1 Data (CSV)
